# Coronary micro-computed tomography angiography in mice

**DOI:** 10.1038/s41598-020-73735-4

**Published:** 2020-10-08

**Authors:** Stefan Sawall, Jan Beckendorf, Carlo Amato, Joscha Maier, Johannes Backs, Greetje Vande Velde, Marc Kachelrieß, Jan Kuntz

**Affiliations:** 1 German Cancer Research Center (DKFZ), X-Ray Imaging and CT, Heidelberg, 69120 Germany; 2 Medical Faculty, Ruprecht–Karls–University Heidelberg, Heidelberg, 69120 Germany; 3 University Hospital Heidelberg, Molecular Cardiology and Epigenetics (Internal Medicine VIII), Heidelberg, 69120 Germany; 4 German Centre for Cardiovascular Research (DZHK), Partner Site Heidelberg/Mannheim, Heidelberg, Germany; 5 Department of Physics and Astronomy, Ruprecht–Karls–University Heidelberg, Heidelberg, 69120 Germany; 6 Department of Imaging & Pathology/ MoSAIC, Faculty of Medicine, KU Leuven, Leuven, Belgium

**Keywords:** Cardiovascular diseases, Biomedical engineering, Imaging techniques, Preclinical research, 3-D reconstruction, X-ray tomography

## Abstract

Coronary computed tomography angiography is an established technique in clinical practice and a valuable tool in the diagnosis of coronary artery disease in humans. Imaging of coronaries in preclinical research, i.e. in small animals, is very difficult due to the high demands on spatial and temporal resolution. Mice exhibit heart rates of up to 600 beats per minute motivating the need for highest detector framerates while the coronaries show diameters below 100 μm indicating the requirement for highest spatial resolution. We herein use a custom built micro–CT equipped with dedicated reconstruction algorithms to illustrate that coronary imaging in mice is possible. The scanner provides a spatial and temporal resolution sufficient for imaging of smallest, moving anatomical structures and the dedicated reconstruction algorithms reduced radiation dose to less than 1 Gy but do not yet allow for longitudinal studies. Imaging studies were performed in ten mice administered with a blood-pool contrast agent. Results show that the course of the left coronary artery can be visualized in all mice and all major branches can be identified for the first time using micro-CT. This reduces the gap in cardiac imaging between clinical practice and preclinical research.

## Introduction

Over the last decades micro-computed tomography (micro-CT) has emerged as a valuable tool in preclinical and clinical research. Classic table-top ex-vivo micro-CT systems provide spatial resolutions of up to 1 μm but do not allow for the acquisition of moving structures observed in living preclinical specimens due to long measurements and long detector integration times^1,2^. Mice, being a popular specimen in preclinical research, show heart rates of up to 600 beats per minute (bpm) and respiratory rates of up to 300 respirations per minute (rpm). For example, to visualize lung motion and equidistantly sample the respiratory cycle using five non-overlapping respiratory phases, the detector integration time must not exceed 40 ms which usually cannot be met by classic table-top systems. Currently available in-vivo micro-CT systems on the other hand provide detector integration times of up to 10 ms, in contrast to ex-vivo systems with integration times in the order of seconds and minutes^3^. This high temporal resolution is only possible at the expense of spatial resolution, i.e. the spatial resolution for in-vivo scans is typically in the order of 75 μm or worse, and the high temporal resolution can only be achieved by detector binning reading out only a reduced number of pixels. Practically achievable spatial resolution on the other hand is limited by the signal-to-noise ratio per unit dose since dose increases by the fourth power of spatial resolution for a given object and noise level. I.e., an increase in spatial resolution by a factor of 2 requires an increase of radiation dose by a factor of 16 to maintain a constant signal-to-noise ratio. The in-vivo resolution achieved by state-of-the-art micro-CT systems has proven sufficient for a variety of applications, pulmonary imaging and quantification of cardiac output being popular examples^4,5^. It is still limited to larger vessels, e.g.  the aorta, the pulmonary trunk and major branches thereof, and large structures, e.g. cardiac ventricles and atria. However, current preclinical micro-CT technology fails to provide reconstructions of the smallest moving structures with diagnostic image quality, e.g. in case of smallest lung metastases, or fails to visualize desired anatomical structures at all, e.g. in case of coronary arteries in murine models of cardiovascular diseases. While the mouse heart is smaller by a factor of about 12 to 15 compared to a human heart, e.g.  on average the long axis length is about 9 mm and the short axis length is about 4 mm depending on the mouse strain compared to 130 mm and 80 mm in humans, respectively, it maintains a constant proportion to body size^6,7^. The general anatomy is similar to humans and the clinical translatability of mouse models of cardiovascular diseases has been proven, underlining the importance in preclinical research. Major differences in anatomy are found in the superior vena cava and pulmonary veins^8^. The anatomy of coronary arteries in mice is similar to the ones of man, as has been validated using corrosion casts and scanning electron microscopy^9,10^. The left (LCA) and right coronary arteries (RCA) emerge from the respective coronary sinuses. The left coronary trunk branches into the obtuse marginal artery (OMA), similar to the left anterior descending (LAD) in humans, and the left circumflex (LCX). The former courses the left ventricular wall to the apex in an oblique fashion and variably branches into smaller vessels. The latter courses parallel to the left atrioventricular groove, giving rise to the left marginal artery, and terminates near the crux cordis. The RCA courses parallel to the right atrioventricular sulcus and supplies the right side of the heart. It gives rise to several variable branches, some of them bound for the apex. Diameters of LCA and RCA have been estimated to about 400 μm and 260 μm using Ultrasound and 9.4 T MRI, respectively, while the average coronary diameter was estimated as 160 μm in ex-vivo studies^10–13^. This discrepancy between in-vivo and ex-vivo estimates might be caused by sample shrinkage due to tissue preparation in the ex-vivo studies or less likely by the finite integration time, residual motion and consequent blurring of moving structures in the in-vivo experiments. While motion velocities for coronary arteries are well known in humans, in particular the right coronary artery moves significantly faster compared to the left coronary^14^, such values seem not to be available in mice. However, given the striking resemblance in cardiac anatomy, one might assume that similar conditions apply. While coronary computed tomography angiography (CCTA) is a common tool in clinical practice providing time-resolved images of the complete coronary tree including all relevant branches, a preclinical equivalent is not yet available for a variety of reasons. First, the high cardiac and respiratory rates pose tremendous demands on the imaging hardware as simultaneously high temporal and high spatial resolution is required to account for the small, rapidly moving coronaries. Second, data acquisition in a single heartbeat, as often performed in clinical routine, is extremely challenging in murine models due to the low available detector framerates and the inability of voluntary breath hold. Furthermore, long scan times require the use of blood pool agents, i.e. contrast media that circulate in the blood for a prolonged time, as the high metabolic rates of small animals result in a quick wash-out of standard clinical contrast agents introducing data inconsistencies and consequently artifacts in the reconstructed images^15,16^. Fourth, prospective gating methods are not readily available in cardiac micro-CT imaging, albeit a few experimental methods have been published recently requiring dedicated hardware, and hence large amounts of data have to be acquired and retrospectively sorted for desired cardiac and respiratory phases, usually increasing the administered radiation dose to a level that seems inappropriate for longitudinal studies^17–19^. While many methods have been developed to reduce radiation dose administered in micro-CT examinations by orders of magnitude, cardiac micro-CT is still inferior to clinical cardiac imaging in terms of spatial and temporal resolution compared to the size of anatomical structures and motion velocities under investigation^15,16,20,21^. Current capabilities of in-vivo cardiac micro-CT are limited to the visualization of large cardiac structures, i.e. of atria, ventricles and major supplying and draining vessels, the quantification of ventricle size, e.g. in models of pulmonary hypertension, and consequently the quantification of cardiac function, e.g. the left ventricular ejection fraction^4,5,22–24^. Recently, experimental time-resolved dual-energy imaging techniques have been introduced^25^ and a single method for cardiac perfusion imaging became available^26^ by using a novel contrast injection technique^27^. Furthermore, several contrast agents have been proposed that selectively accumulate in healthy myocardium. It was shown in models of myocardial infarction, inflicted by a ligation of the left anterior descending artery (LAD), that these contrast agents allow for a differentiation between viable and infarcted tissue^25,28^. The LAD, or any coronary artery for that matter, could never be visualized in these experiments. We herein aim at illustrating that coronary imaging in mice is possible using preclinical in-vivo micro-CT.

## Materials and methods

### Animal preparation and handling

All animal experiments are approved by the legal authority on animal welfare (Regierungspräsidium Karlsruhe, G-256/15) and were in accordance with the guidelines issued by the Federation of European Laboratory Animal Science Associations (FELASA)^29^. Prior to any micro-CT examinations, ten C57BL/6J mice were placed in an acrylic glass box and anesthesia was delivered via inhalation of isofluorane ($2\,\% +\text {O}_2$). As soon as the desired depth of anaesthesia was reached, verified by an absence of the paw and corneal reflex, a catheter was inserted into the lateral tail vein. This catheter was used to deliver $100\,\upmu \text {L}$ of a blood pool contrast agent (ExiTron nano 12000, nanoPET Pharma GmbH, Berlin, Germany) followed by a $25\,\upmu \text {L}$ saline chaser to ensure that all contrast media enters the circulation. The animals were transferred to the mouse bed in the system were anaesthesia was ensured by a constant flow of isoflurane. Furthermore, the animals were monitored during the measurements using a camera to ensure their well-being. As physiological signals, i.e. gating signals for cardiac and respiratory motion, will be deduced intrinsically from the acquired data, an equipment of the animals with ECG-electrodes or a pneumatic pillow is not required^30,31^.

**Figure 1:**
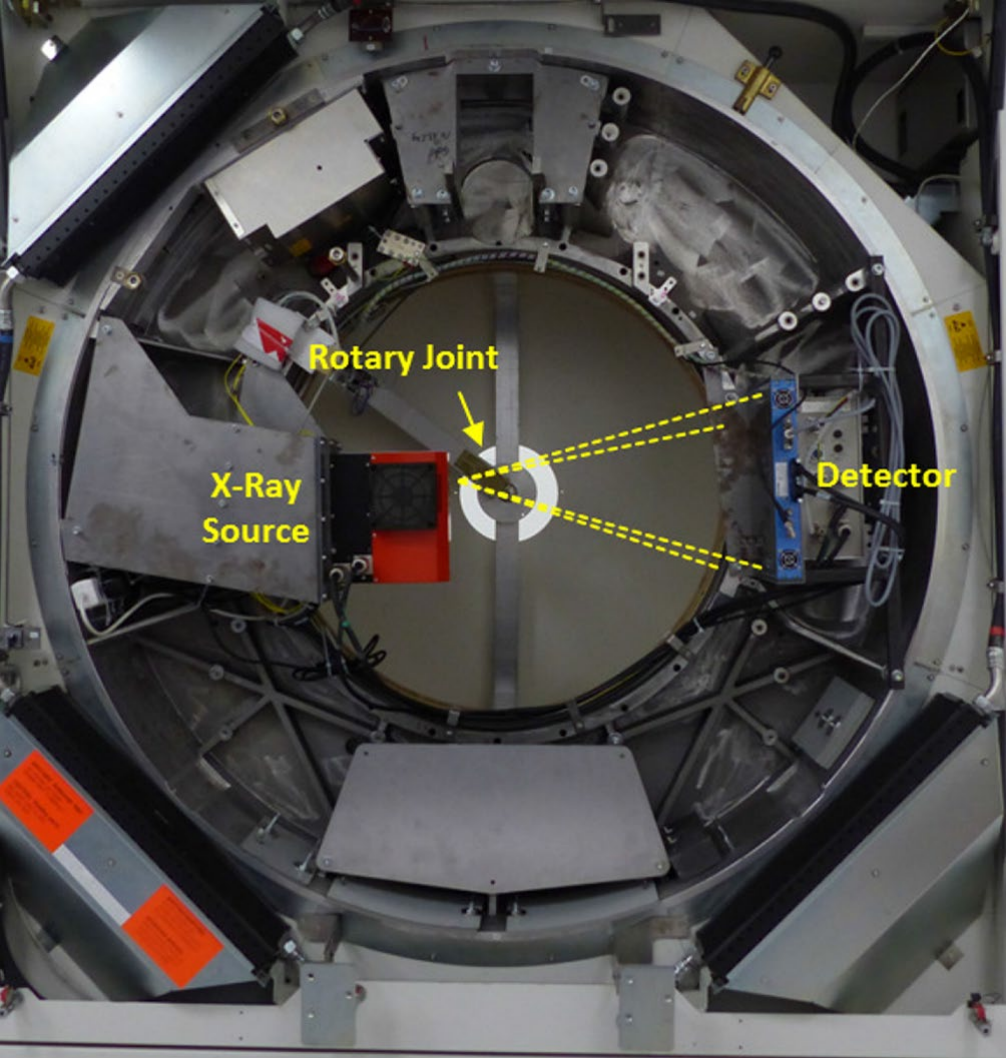
Photo of the used prototype micro-CT system. The yellow dashed lines indicate the cone-beam geometry of the system formed by a microfocus X-ray source and a high-speed X-ray detector. Data are transmitted using a rotary joint located in the back of the gantry.

**Figure 2:**
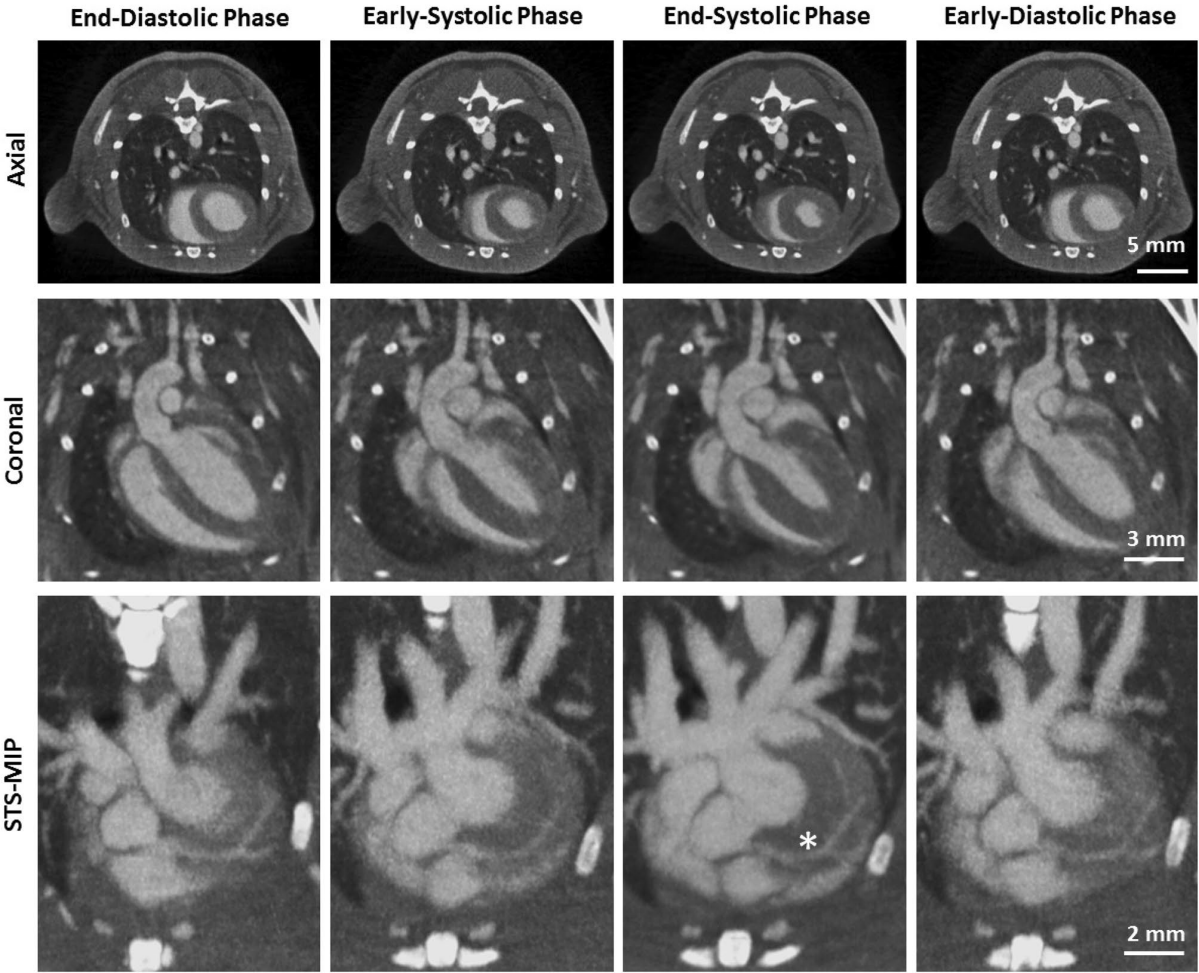
Phase-correlated reconstructions of a mouse showing different cardiac motion states (columns). The first row illustrates axial slices, the second row coronal slices and the third row a sliding thin slab-maximum intensity projections (STS-MIP). Gating parameters are chosen as described in the text. The asterisk marks the first bifurcation of the left coronary artery. ($C=300\,\text {HU}, W=1500\,\text {HU}$).

### Imaging system and scan protocol

All imaging studies have been performed using a custom built micro–CT system. In brief, this system is based on a refurbished clinical CT gantry (Siemens Volume Zoom, Siemens Healthcare, Forchheim, Germany) allowing for a rotation speed of up to $0.5\,\text {s}$ per revolution and providing the required dimensions to house all components, i.e.  X-ray source and detector, with the desired geometric alignments. In particular, the microfocus transmission X-ray source (L10951, Hamamatsu Photonics K. K., Shimokanzo, 195 Iwata City, Japan) using a tungsten target is mounted with a source-isocenter distance of $R_{\text {F}}=90\,\text {mm}$. The CMOS flat detector (Dexela 2923 MAM, Perkin Elmer, A Varex Company, Salt Lake City, USA) is mounted with a source-detector distance of $R_{\text {FD}}=590\,\text {mm}$ and provides $3888\times 3072$ pixels with a square pixel size of $74.8\,\upmu \text {m}$. All data from the gantry are transmitted using a rotary joint to the respective control units (see Fig. 1). All experiments have been conducted using a tube voltage of $60\,\text {kV}$ at $50\,\text {W}$, i.e.  using a focal spot size of $80\,\upmu \text {m}$. The detector is operated in $4\times 4$ binning mode with a framerate of $86\,\text {fps}$ or $11.7\,\text {ms}$ integration time, respectively, to account for the high cardiac rates of mice. This setup results in a spatial resolution at 10% modulation of $\text {MTF}_{10\%}=7.5\,\text {lp/mm}$. The gantry is operated using a rotation time of $10\,\text {s}$ per revolution to limit the influence of angular blurring. A single scan is comprised of $5\,\text {min}$ scan time, corresponding to a radiation dose of $5\,\text {Gy}$, as measured in a mouse-sized PMMA cylinder using an ionization chamber (PTW NOMEX, PTW, Freiburg, Germany). However, this overall dose is only used to obtain an almost noise-free ground truth. We will motivate and demonstrate below that coronary arteries in mice can be visualized with administered radiation doses almost suited for longitudinal studies by using only fractions of the acquired data. All gating signals required for the reconstruction of time-resolved data are acquired intrinsically^30,31^. If not noted otherwise, all reconstructions have been performed using four non-overlapping respiratory gating windows with a width of $\Delta r=25\%$ and ten non-overlapping cardiac gating windows with a width of $\Delta c=10\%$.

**Figure 3:**
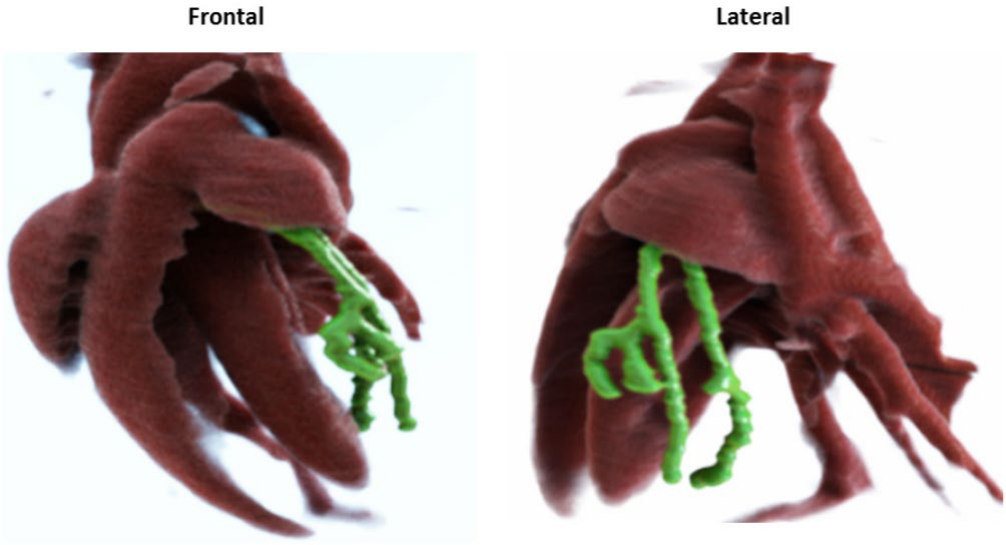
Volume rendering of the left coronary artery (green) and the cardiac anatomy (red) in one of the ten mice. Note that the first bifurcation of the left coronary is covered by the left auricle and cannot be seen in the figure. (Exposure Render 1.1.0^32^, https://github.com/ThomasKroes/exposure-render).

### Image reconstruction and dose reduction

The standard reconstruction of the used micro-CT system is a highly optimized GPU-based implementation of the FDK (RayConStruct-IR, RayConStruct GmbH, Nürnberg, Germany)^33^. In particular, given polychromatic rawdata ${\varvec{q}}$ a standard reconstruction ${\varvec{f}}_\text {Std}$ is obtained as

1 $$\begin{aligned} {\varvec{f}}_\text {Std}={\mathsf {X}}^{-1}{\varvec{q}}, \end{aligned}$$

wherein ${\mathsf {X}}$ is the X-ray transform and ${\mathsf {X}}^{-1}$ denotes a filtered backprojection. While all acquired projections are used in this reconstruction the resulting volume is blurred in regions with motion but is of high image quality elsewhere. To account for motion, a phase-correlated (PC) reconstruction might be used that only backprojects the projections that are within desired motion bins, i.e.  in desired cardiac and respiratory phases:

2 $$\begin{aligned} {\varvec{f}}_\text {PC}={\mathsf {X}}^{-1}_\text {PC}{\varvec{q}}, \end{aligned}$$

wherein ${\varvec{f}}_\text {PC}$ is a phase-correlated volume obtained using a phase-correlated reconstruction ${\mathsf {X}}^{-1}_\text {PC}$^34^. It is well known that ${\varvec{f}}_\text {PC}$ suffers from streak artifacts in low-dose scenarios where only a limited number of projections are available. I.e., in case of a phase-correlated reconstruction only about $2.5\%$ of all acquired data contribute to the reconstruction of a single volume if the afore-mentioned gating settings are used. To overcome this issue, we use a motion estimation and compensation technique proposed recently to reconstruct images with a high temporal resolution and diagnostic quality^20^. In brief, this method is based on the Demons algorithm and estimates motion vector fields between adjacent phases of a phase-correlated reconstruction while ensuring that these vector fields are cyclic, i.e. ensuring that motion forms a closed loop and returns to the same motion state after a complete motion cycle^35,36^. As soon as all required vector fields are estimated, the phase-correlated reconstructions are deformed to match a desired motion phase, in this case a desired cardiac and respiratory phase. Consequently, all deformed volumes are superimposed to obtain a motion-compensated volume ${\varvec{f}}_\text {MoCo}$. As all of the available data are deformed to a desired motion phase, consequently all data contribute to the final reconstruction and thus ${\varvec{f}}_\text {MoCo}$ resembles the low noise level of ${\varvec{f}}_\text {Std}$ while still providing the temporal resolution of ${\varvec{f}}_\text {PC}$. Given this unique property of ${\varvec{f}}_\text {MoCo}$ implies that the radiation dose required to provide volumes with a high temporal resolution is equivalent to the radiation dose required to provide a standard reconstruction if this technique is used. Hence, the motion compensation methods developed in previous publications are an important prior work to enable coronary CTA in small animals. Assuming that all estimated motion vector fields are an accurate representation of the actual motion during the scan, the resulting image ${\varvec{f}}_\text {MoCo}$ exhibits the same spatial resolution as observed in ${\varvec{f}}_\text {PC}$. However, the performance of the Demon’s algorithm or any registration algorithm for that matter is a function of signal-to-noise ratio. Hence, the resulting images might not reach the spatial resolution observed in static scans. Since the structures of interest, i.e. larger vessels and coronary arteries, are highly contrasted by the used blood-pool agent, no significant resolution difference between ${\varvec{f}}_\text {PC}$ and ${\varvec{f}}_\text {MoCo}$ could be observed, as was verified similar to previous publications^20^.

**Figure 4:**
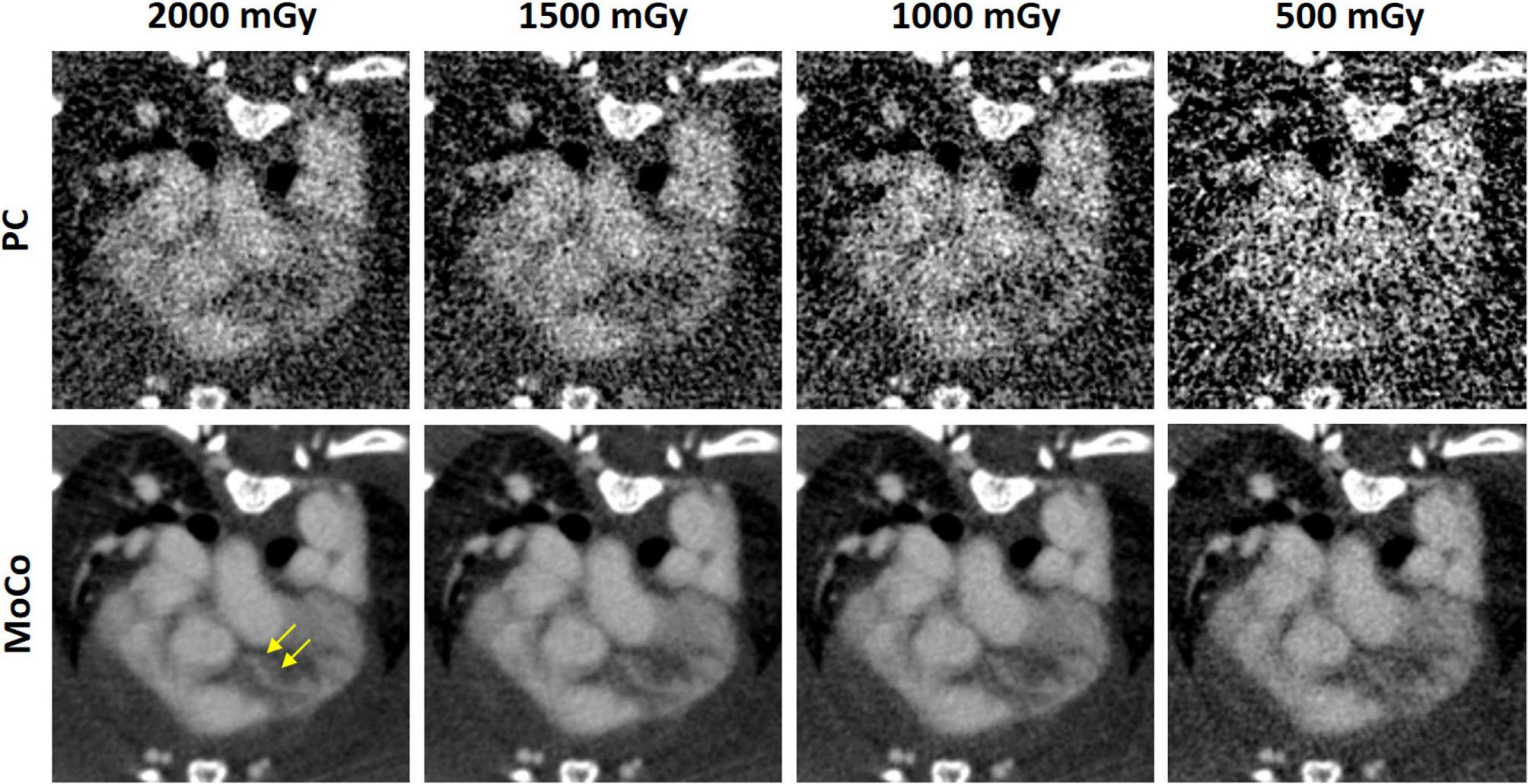
Dose reduction study with radiation dose levels between $2000\,\text {mGy}$ and $500\,\text {mGy}$. The top row shows the results obtained using a conventional phase-correlated (PC) reconstruction and the bottom row illustrates results obtained using a motion compensation (MoCo) method. ($C=300\,\text {HU}, W=1200\,\text {HU}$).

### Data postprocessing

Given the fact that the motion compensation results in an image quality equivalent to a standard reconstruction in terms of image noise, we apply an anisotropic multi-dimensional adaptive tensor-based filter (TBAF) to the volumes in a postprocessing step^37^. In brief, this filter computes a local orientation tensor, estimates Eigenvectors and Eigenvalues of this tensor and filters in the direction corresponding to the largest Eigenvalue while adapting the filter strength to local noise properties. It thus preserves spatial resolution and significantly reduces image noise. While this filter does not require any user interaction, it can furthermore be shown that the results overcome the staircasing artifacts often observed using a bilateral filter^38^.

## Results

### Coronary CT angiography in mice

Figure 2 shows reconstructions obtained in one of ten mice using an administered radiation dose of 5 Gy employing a phase-correlated reconstruction. Columns in the figure show different cardiac phases covering the complete cardiac cycle. i.e., the first column illustrates a reconstruction obtained in an end-diastolic phase, hence the blood-filled left and right ventricles, the second and third row show reconstructions obtained in early and and late systole, respectively, and the fourth column shows reconstructions in an early diastolic phase. The first row shows axial slices in the respective cardiac phases while the second row shows coronal slices. Similar to ventricles, the volumes of the atria illustrate the cardiac states. While they are contracted in diastole, i.e. mitigating blood flow to the ventricles, they are relaxed in systole and filled with blood. Furthermore, the diameter of the pulmonary trunk increases as blood is ejected into the pulmonary circulation in systole and its diameter decreases as blood pressure drops in diastole as can be seen in the coronal slices. A visualization of coronary arteries in either axial or coronal slices, however, is difficult given the high curvature of these vessels. The bottom row of Fig. 2 shows a STS-MIP (sliding thin slab- maximum intensity projection) in the longitudinal direction. The course of the left coronary artery can be clearly observed in all cardiac phases. It emerges at the respective coronary sinus in the ascending aorta and courses along the left side of the heart. Its first bifurcation gives rise to the LCX and OMA. Consequent branches of these arteries supplying the myocardium are visualized in the figure as well. As expected, the left coronary can be best visualized in diastole in terms of visibility as the motion velocity is significantly smaller in this cardiac phase compared to the systole. Considering the high radiation dose of 5 Gy used to acquire the images in this figure, the left coronary artery and its branches can easily be recognized using the phase-correlated reconstruction shown here. Hence, given the vast amount of data available, PC reconstructions do not suffer from streak artifacts as often observed in low-dose scenarios. Unlike the left coronary artery, the right coronary artery could not be visualized in this mouse nor in any other of the ten mice which is most likely attributed to its high velocity.

While Fig. 2 only shows a fraction of the left coronary artery, Fig. 3 presents a volume rendering (Exposure Render 1.1.0^32^, https://github.com/ThomasKroes/exposure-render) of the LCA in one of the mice under investigation. In particular, the left coronary and its branches are visualized in green while all other vessels and vasculature are visualized in red. The vasculature was obtained by a simple thresholding as the contrast-enhanced structures can be accurately segmented from reconstructed data. To obtain a volume of the coronary artery, its centerline was automatically determined and a volume with a constant radius of 1 mm was generated around this centerline. Hence, the diameter of the coronary artery shown in Fig. 3 does not reflect accurate anatomical findings but was artificially increased for display purposes. In particular, the figure illustrates that the left coronary can be followed to the apex of the heart. Note that the first bifurcation of the artery as marked by an asterisk in Fig. 2 is covered by the left auricle and cannot be seen in the figure. Hence, the major branches seen in the figure are the OMA and the LCX.

**Figure 5:**
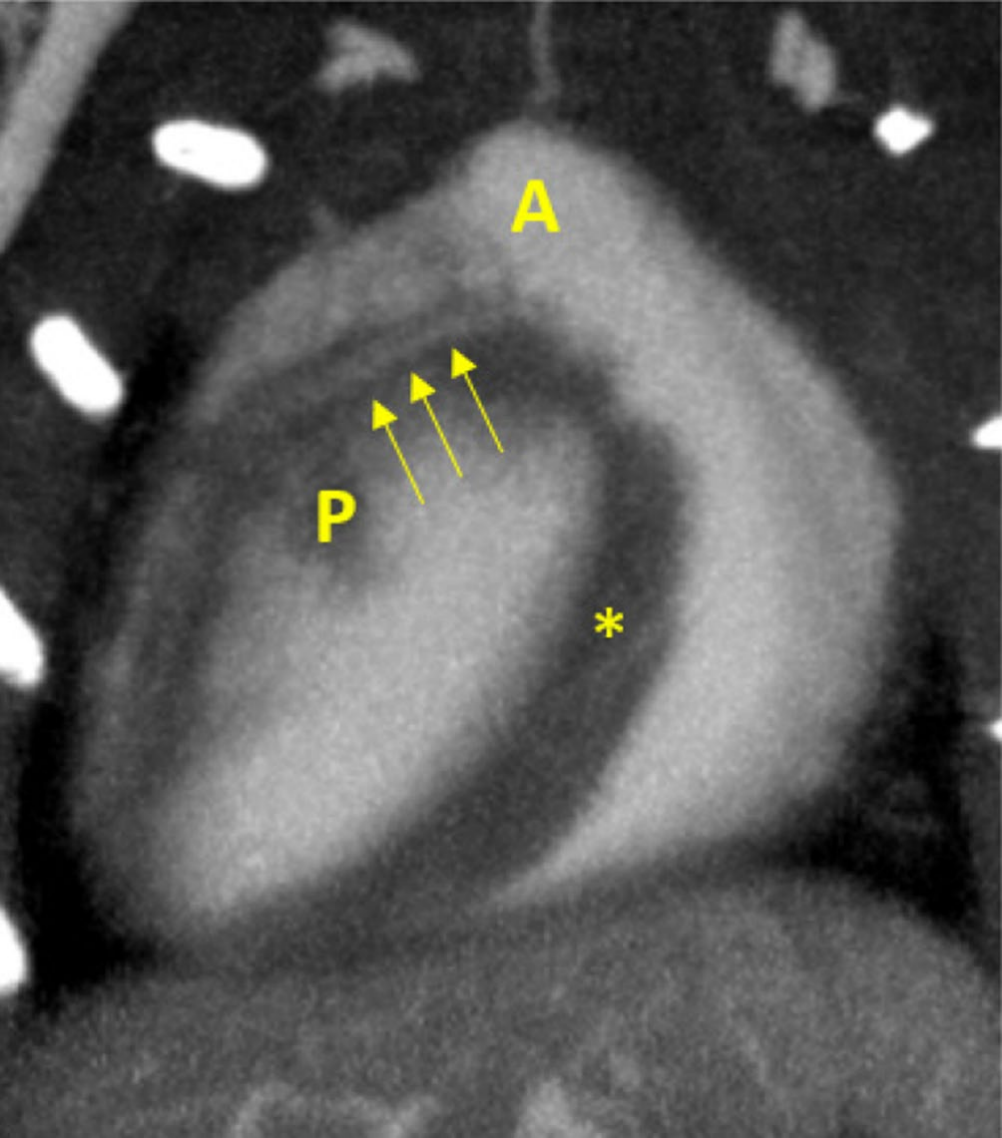
Coronal STS-MIP of another mouse showing the left coronary artery (yellow arrows) emerging from its ostium at the aorta (A). Unenhanced regions in the left ventricle are papillary muscles (P). A small vessel seems also visible in the vicinity (*) of the right ventricle. ($C=300\,\text {HU}, W=1200\,\text {HU}$).

In general, the left coronary trunk could be visualized in all mice. This is exemplary illustrated in Fig. 5 (yellow arrows) for another mouse. The first bifurcation into the obtuse marginal artery and the left circumflex artery could be visualized in all mice as well. In 60% of all cases, the former could be followed to the apex of the heart and several smaller branches could be visualized. In all cases, the first branch of the left circumflex artery into the left marginal artery could be identified as well as a varying number of consecutive smaller branches of both vessels. Unfortunately, the right coronary artery could not be visualized in any of the mice.

### Dose considerations

While the radiation dose used to obtain the reconstructions shown in Fig. 2 is in the range of the lethal radiation dose $LD_{50}$, i.e. resulting in the death of about 50% of the exposed animals if the whole body were be irradiated, this imaging procedure prohibits longitudinal studies. Even if the animals were to survive, severe metabolic interferences, e.g. blood count alterations, were to be expected. Figure 4 illustrates the possible dose reduction achievable with the methods described before. In particular, the figure illustrates images achieved with radiation dose levels between $2000\,\text {mGy}$ and $500\,\text {mGy}$ obtained using the conventional phase-correlated reconstruction (top row) and the motion compensation (MoCo) method (bottom row). Yellow arrows indicate the left coronary artery after its emergence from the respective coronary sinus. Image noise increases with decreasing radiation dose. However, the LAD can be identified in the MoCo reconstructions up to a dose level of about $1000\,\text {mGy}$ while it cannot be identified in any of the PC reconstructions. This is caused by the high noise level in these reconstructions and a degradation of image quality by streak artifacts for lower dose levels and lower projection counts.

## Discussion and conclusion

We herein illustrated that coronary imaging is possible using preclinical in-vivo micro-CT. While the left coronary artery and its main branches and sub-branches could be observed from the coronary sinus to the cardiac apex in all mice, the right coronary could not be visualized with sufficient quality. Similar to clinical CT, this might be caused by the higher motion velocity of the right coronary artery compared to the left coronary. A verification of this hypothesis might be topic of future research and could be conducted as soon as detectors with higher framerates become available or by using other imaging modalities, e.g. ultrasound imaging. The spatial resolution of the used system and scan protocol is in the order of $70\,\upmu \text {m}$. Ex-vivo studies illustrated that the size of murine coronary arteries is in the same order of magnitude at the ostia and rapidly decreases towards the cardiac apex. This seems to contradict the possibility for coronary imaging in small animals within the used system. We hypothesize that this apparent discrepancy might be explained by two mechanisms. First, the results obtained in ex-vivo studies might be inaccurate due to the preprocessing of the tissue samples. e.g., it is well known from histological staining that samples might shrink depending on the methods used for tissue preparation. Second, the used cardiac gating windows of $\Delta c=10\,\%$ might introduce a blurring as it integrates over the respective fraction of the cardiac cycle. The latter claim could be verified by acquiring a vast amount of data ignoring dose constraints for once, consequently allowing for image reconstruction using smaller gating windows. This might be a topic of future research as it might further the understanding of cardiac motion in small animals. Furthermore, the available spatial resolution of about $70\,\upmu \text {m}$ is similar to previous experiments^15^. However, no coronary arteries could be observed in these experiments, potentially due to the low detector framerate of only $25\,\text {fps}$ compared to the framerate of $86\,\text {fps}$ used in the present study. This indicates that standard micro-CT systems might be able to visualize coronary arteries in terms of spatial resolution but lack the required temporal resolution. A dose reduction study illustrated that coronary imaging in small rodents is possible with dose levels not yet allowing for longitudinal studies with a multitude of measurements. Given the prototype nature of the used system indicates that future research might result in further dose reductions eventually allowing for longitudinal studies similar to what was observed after the introduction of cardiac CTA in clinical practice. i.e., clinical CCTA exhibited high dose levels of up to $30\,\text {mSv}$ upon its introduction into clinical practice^39^. Advances in technology, however, allowed for a significant reduction of the required radiation dose to $1\,\text {mSv}$ or even lower in the years that followed^40^. We expect a similar reduction of radiation dose for preclinical murine CCTA examinations in the near future. This, for example, could be achieved by exploiting the small pixel size and high framerates of photon counting detectors. i.e., the detector used in this study is operated in a $4\times 4$-binning mode to achieve the desired framerate. However, it is known from theory and recent publications that acquisitions with smaller pixels results in lower image noise when reconstructed to the same spatial resolution as an acquisition with larger pixels acquired at the same dose^41–43^. Since future photon-counting detectors provide high framerates even without pixel binning, this effect could potentially be used to decrease dose. Considering the small size of cardiovascular structures and the fast motion velocities in small animals, the reconstructions provided herein do not yet match the high image quality of clinical CCTA. However, the proposed methodology decreases the gap between preclinical and clinical imaging and might boost preclinical research in small animal models of cardiovascular diseases.
